# Eukaryogenesis: Did an Oxidative Crucible Result in Misleading Bioinformatic Analyses?

**DOI:** 10.1002/bies.70115

**Published:** 2026-02-17

**Authors:** Dave Speijer

**Affiliations:** ^1^ Medical Biochemistry Amsterdam UMC Location University of Amsterdam Meibergdreef 9 Amsterdam The Netherlands

## Abstract

Recently, Nature published a large‐scale analysis (“Dated gene duplications elucidate the evolutionary assembly of eukaryotes” by Christopher Kay and co‐workers) that seems to put an end to symbiogenic models for eukaryogenesis. They state that the pre‐mitochondrion arrives late, after practically all of the signature eukaryotic characteristics have evolved independently. However, this conclusion is based on reconstructed timescales for the gene duplications allowing these crucial eukaryotic cell functions. The reconstruction might be fundamentally flawed, because enhanced internal ROS formation upon endosymbiont entry would lead to both high mutation rates and strong selection for antioxidant as well as repair functions. As the endosymbiont had co‐evolved with molecular oxygen, while the archaeal host had not, a phylogenetic analysis might misconstrue the higher rate of change in the host as indicative of much longer timescales for host gene duplications.

## Graphical abstract

Recently, Nature published a timed gene duplication analysis favoring gradual models for eukaryogenesis. However, high initial mutation rates due to endogenous ROS formation by the OXPHOS system of the future mitochondrion and ensuing positive selection for countermeasures might strongly inflate branch lengths in such analyses. Ruling out symbiogenesis seems premature.

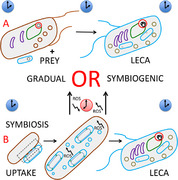

## 1 Discarding Symbiogenic Models is Premature

The highly impressive recent article “Dated gene duplications elucidate the evolutionary assembly of eukaryotes” by Kay et al. [1] makes some sweeping claims: “…results allow us to reject mitochondrion‐early scenarios of eukaryogenesis…”, instead proving the existence of a eukaryotic host cell that “…was already very complex before mitochondrial endosymbiosis, including an elaborated cytoskeleton, membrane trafficking, endomembrane, phagocytotic machinery, and a nucleus…” [1].

The temporal occurrence of events leading to the last common ancestor of the eukaryotes (LECA) population, appearing around 2 billion years ago is notoriously hard to reconstruct and the authors might very well be right regarding both this sequence of events and the timing of their incidence. However, they infer a lot based on only one type of analysis (in effect, on the reconstructed timescale of the gene duplications which would allow many of the crucial eukaryotic cell functions). Importantly, the authors’ conclusions also seem to settle other disputes, e.g., whether the evolution of the eukaryotic complexity depended on efficient ATP generation by oxidative phosphorylation (OXPHOS) on extended internal membranes [2, 3] or not [4]. According to them, it did not, as seen from the relative timeline of their reconstruction: microtubules; ER/golgi; nucleus; endosome/lysosome; phagocytosis, with only the replacement of archaeal membranes by bacterial ones and the preprotein import system likely coevolving with subsequent mitochondrial endosymbiosis [1]. Only meiotic sex [5, 6, 7] and peroxisomes [8, 9] (which the authors do not mention) could be the result from symbiogenic interactions, according to this timeline. So, are we indeed looking at the final demise of strong symbiogenic models [10, 11]?

**FIGURE 1:**
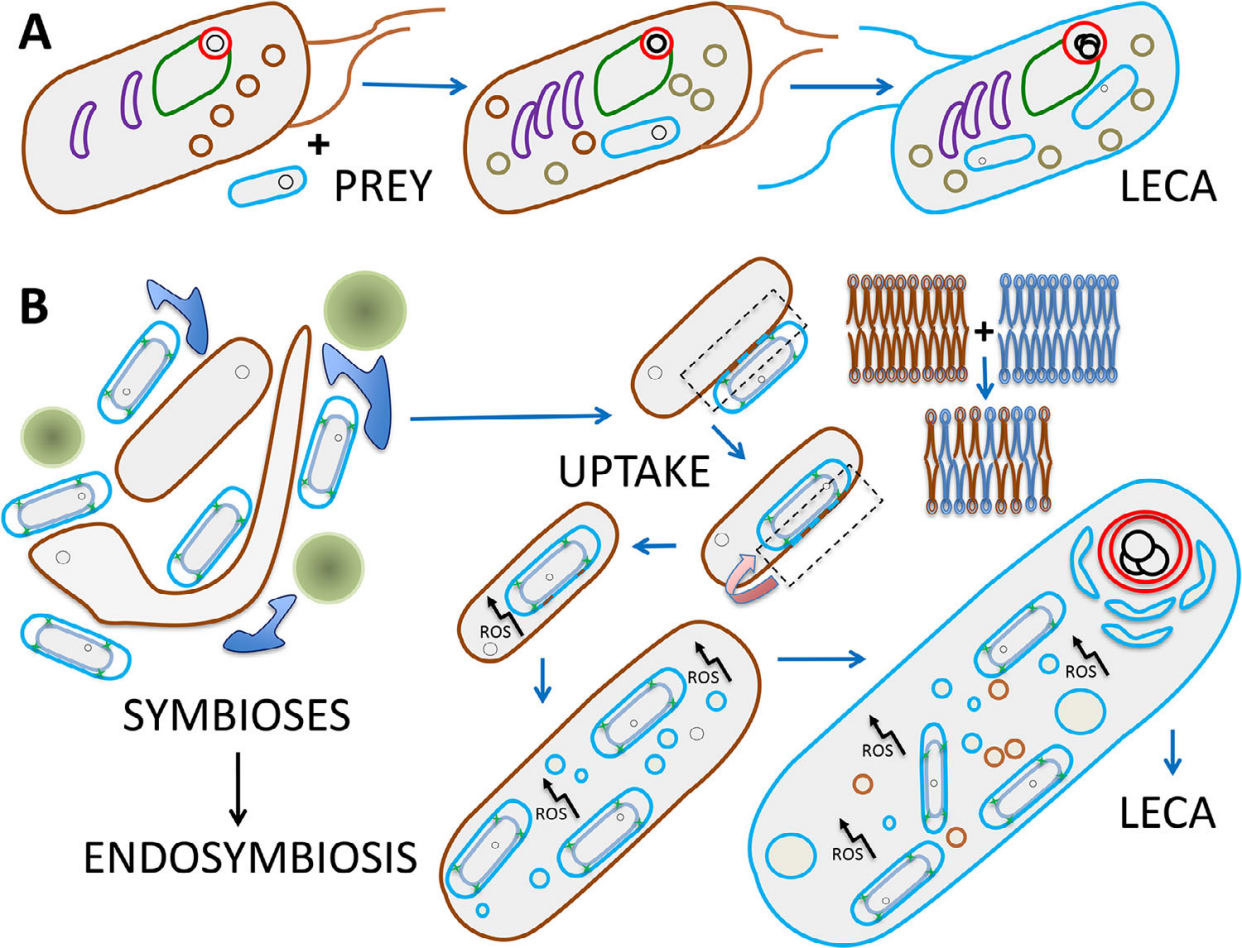
Phagocytotic uptake or (accidental) engulfment? (A) Highly schematic depiction of a complex pre‐eukaryote phagocytosing an alpha‐proteobacterium, leading to LECA, as deduced in [1]. (B) An *Asgard archaeon* living in a complicated ecosystem with other prokaryotes (here consisting of three different species) entangles an alpha‐proteobacterium, with most (all?) of the internal complexity arising from symbiogenic interactions between the two organisms, triggered by endogenous ROS formation. Symbiosis depiction inspired by figure 3 h in [12]. Entry follows temporary mixing of the archaeal and the bacterial outer membrane [28]. Archaeal membranes—brown; bacterial membranes—blue; nuclear membranes (possibly derived from outer membrane vesicles in B [26])—red; ER—green; Golgi—purple.

We might still hesitate, because the results obtained by Kay et al. make one persistent riddle bigger. We are confronted with what we might playfully describe as the ‘FERPI paradox’: where are the descendants of these First Eukaryotic Roving Phagocytotic Insurgents (FERPIs) that never gained the endosymbiont (the so‐called ‘Archezoans’ of old)? Because with all the recent discoveries about *Asgard archaea*, we are witnessing a somewhat strange phenomenon. The genomic implications are happily embraced, but outcomes of Asgard cultivation experiments are overlooked. Genetically complicated Asgardians have been cultivated; they need other prokaryotes in their cultivation media (they form part of complicated syntrophic networks), but have *no discernible organelle‐like internal structure*, although forming striking extrusions possibly optimizing metabolite exchange [12, 13]; see also Figure 1.

Thus, though according to [1] complex pre‐eukaryotes were present for a long time, they seem to have left only descendants which have (or used to have) mitochondria. A previous explanation for their absence, that they were outcompeted by the much more efficient LECA and its descendants, does not fit well with the now heavily reduced contribution of the mitochondrion. But if we suppose instead that such an intermediate organism never existed, how to explain the duplication timeline? We have no reason to doubt the association of gene duplications with the subsequent (relatively direct?) emergence of their new functions, even though some gene products forming parts of molecular machines can, on occasion, unpredictably exchange between completely different molecular machinery [14]. That only leaves the timing itself. The dangers of phylogenomic analyses, especially when applied to eukaryogenesis are discussed in [3, 15]. I quote: “However, eukaryogenesis must have involved significant shifts in evolutionary constraints on proteins as they adopted new functions.” [15]. Of course, the authors, “using a relaxed molecular clock” are sure that they compensate for all possible shifting evolutionary constraints.

Here I have to introduce a very important publication which might indicate what kind of really extraordinary evolutionary constraint might still have been missed. In 2015, van der Sluis and co‐workers showed that upon uptake of the endosymbiont, approximately 75 (!) novel subunits were added to mitoribosomes and OXPHOS complexes, initially *just to compensate mutationally destabilized* mitochondrially encoded components. No such process occurred with plastids (taken up later, by full‐fledged eukaryotes) [16, 17]. If we look at figure 1B in this light, such instances of mostly ‘useless complexification’ should also have happened in the ‘host’, and if anything, could have given rise to even more, and more rapid, damage and change, because *its genome and cellular defences had not been shaped by a previous aerobic lifestyle*; compare [18]. The recent findings of [1] fit surprisingly well with a relatively short period of highly abundant mutation inducing reactive oxygen species (ROS) formation and strong selection for repair and compensatory genome changes. Crucially, such an intense period of enhanced internal ROS formation upon uptake would almost cruelly mislead bioinformaticians to grossly overestimate the absolute time of gene duplications, though their relative timing might still be accurate.

Speaking of ‘an intense period of enhanced internal ROS formation’, how strong could the oxidative stress expected to have been upon this mitochondrial integration into a previously non‐O_2_‐adapted host? Answering this question, even qualitatively, is fraught with difficulties. However, some observations might illustrate the severity of the challenge. How much of the O_2_ used by OXPHOS ends up in the form of ROS? An influential review [19] compares the often mentioned ∼2% of total oxygen consumed by mitochondria under physiological conditions, with ROS production in mitochondria with disabled antioxidant systems. Values obtained with *isolated* heart mitochondria fluctuated between 0.25% and 11% with different species of animals and respiration rates [20]. Other studies (such as [21]) point at lower estimates (∼0.2%) under physiological conditions. The complexity of mitochondrial ROS formation, with different, alternating, OXPHOS substrates is such that estimates about its production by an acquired alpha‐proteobacterium must be very uncertain [19, 22, 23]. However, even the lowest estimate has one of every 500 molecules of O_2_ becoming a source of possible mutations. What that meant for the engulfed bacterium *that had coevolved with O_2_* is illustrated by the findings of van der Sluis et al. [16] mentioned above. Thus, highly increased mutation rates in the ‘unprepared’ host together with strong positive selection for compensatory developments, seem sufficient to explain possible branch length inflation in the duplication‐based phylogenetic analyses.

If we return to the article [1], and look at the figures and data associated with the duplications (including in their supplementary information) initial ‘useless complexification’ associated with compensation as well as proteins involved in DNA repair and prevention of oxidative damage seem to be abundantly present. Alphaproteobacterial‐origin duplications gave rise to surprising paralogues now functioning in the nucleus. Among the earliest are an RNA methylase (METTL4) with functional links to the Lsm and Sm complexes (see below) [24], type Y family DNA polymerases (*low‐fidelity DNA polymerases crucial for cellular survival following DNA damage*), and *mismatch repair* proteins. These duplications possibly even slightly predate those enabling the development of the preprotein import system [1]. The results of host archaeal duplication instances include: three, each more complex, RNA polymerases; DNA polymerase complexes; several DNA repair components; Histones (which can protect DNA from oxidative damage, though it has to be said that they are found in many archaea, and are also understood as the result of coevolution with genome expansion and/or increased regulatory complexity [25]); Proteasome components (involved in breakdown of damaged proteins); and the Lsm and Sm complexes, amongst others involved in splicing (compare the evolution of the mitoribosomes). Even the relatively early completion of a nuclear envelope (see timeline above), e.g., from bacterial OMVs [26], would make sense in the light of genome protection against abundant internal ROS challenges (see Figure 1B) [11]. Of course, the duplications mentioned here allowed larger genome sizes, and ever‐increasing cellular complexity with further compartmentalization during eukaryogenesis, which most likely quickly gave rise to positive selection as well.

## 2 Further Considerations and Future Research

The model favored by Kay et al. faces a further difficulty, the challenging step from a phagocytotic predator to a syntrophic partner, which does not occur upon accidental uptake, because there is a pre‐existing syntrophic relationship (again, see Figure 1B). Modern eukaryotes still show signs of an ancestral syntrophy between Asgard‐archaeal host cells and alphaproteobacterial endosymbionts, e.g., in the eukaryotic genes for central carbon metabolism [11, 27]. This brings me to the last issue: the question regarding the importance and possible role of molecular oxygen in eukaryogenesis. Here Kay et al. speculate about “a complex archaean host cell in oceans that would have remained largely anoxic for more than a billion years” [1]. I do not want to revisit the ongoing discussion about whether mitochondrial oxidative phosphorylation was indispensable for the development of eukaryotic complexity or not, and only refer to [3] and references therein; see also [2, 4]. But, whatever the preliminary stages, many LECA characteristics show the crucial influence of molecular oxygen and associated ROS formation; see [17] and references therein.

I am well aware that a large majority of researchers working in the field is convinced that model A of Figure 1 is basically correct, especially considering the much more ‘accidental’ character of model B. But the fact remains that the endosymbiont co‐evolved with molecular oxygen and ROS, while the archaeal host did not, and this observation indicates that phylogenetic analyses could be prone to systematic errors. So how would model B be laid to rest? First of all, looking for new Asgard representatives and the (difficult) ongoing cultivation experiments could definitively settle the matter, by identifying an archaeon with a nucleus and phagocytotic capabilities (i.e., taking care of the FERPI paradox, described above). Secondly, maybe clever bioinformatic algorithms could distinguish between the slow and steady developments envisioned in [1], as opposed to a more recent, rapid burst of *oxidative* DNA damage and strand breaks with concomitant strong selection explaining the duplication patterns observed, in the future. Finally, though a long shot, could observing prolonged co‐cultivation of Asgard representatives and alpha‐proteobacteria make the symbiogenic model somewhat more likely instead?

## Author Contributions

The author takes full responsibility for this article.

## Conflicts of Interest

The author declares no conflicts of interest.

## Data Availability

Data sharing not applicable to this article as no datasets were generated or analyzed during the current study.
